# CHCHD2 and CHCHD10 regulate mitochondrial dynamics and integrated stress response

**DOI:** 10.1038/s41419-022-04602-5

**Published:** 2022-02-16

**Authors:** Yu Ruan, Jiaqiao Hu, Yaping Che, Yanyan Liu, Zhenhuan Luo, Jin Cheng, Qi Han, He He, Qinghua Zhou

**Affiliations:** 1 The Sixth Affiliated Hospital of Jinan University, Jinan University, Dongguan, Guangdong 523560 China; 2 The Biomedical Translational Research Institute, Faculty of Medical Science, Jinan University, Guangzhou, Guangdong 510632 China; 3 Department of Anatomy and Physiology, Shanghai Jiao Tong University School of Medicine, Shanghai, 200025 China; 4 Shanghai Center for Brain Science and Brain-Inspired Technology, Shanghai, 201210 China; 5 Department of Anesthesiology, Renmin Hospital of Wuhan University, Wuhan, Hubei 430060 China

**Keywords:** Stress signalling, Mitochondria

## Abstract

Mitochondrial dysfunction is becoming one of the main pathology factors involved in the etiology of neurological disorders. Recently, mutations of the *coiled-coil-helix-coiled-coil-helix* domain containing 2 (CHCHD2) and 10 (CHCHD10) which encode two homologous proteins that belong to the mitochondrial CHCH domain protein family, are linked to Parkinson’s disease and amyotrophic lateral sclerosis (ALS)/frontotemporal dementia (FTD), respectively. However, the physiological and pathological roles of these twin proteins have not been well elaborated. Here, we show that, in physiological conditions, CHCHD2 and CHCHD10 interact with OMA1 and suppress its enzyme activity, which not only restrains the initiation of the mitochondrial integrated response stress (mtISR), but also suppresses the processing of OPA1 for mitochondrial fusion. Further, during mitochondria stress-induced by carbonyl cyanide m-chlorophenylhydrazone (CCCP) treatment, CHCHD2 and CHCHD10 translocate to the cytosol and interacte with eIF2a, which attenuates mtISR overactivation by suppressing eIF2a phosphorylation and its downstream response. As such, knockdown of CHCHD2 and CHCHD10 triggers mitochondrial ISR, and such cellular response is enhanced by CCCP treatment. Therefore, our findings demonstrate the first “mtISR suppressor” localized in mitochondria for regulating stress responses in mammalian cells, which has a profound pathological impact on the CHCH2/CHCH10-linked neurodegenerative disorder.

## Introduction

Mitochondria are double membrane-bound subcellular organelles well-known for producing ATP and controlling metabolism. Mitochondria are highly dynamic organelles that undergo continual cycles of fusion and fission to maintain their shape, structure, and function. Fusion of mitochondrial outer (OM) and inner membranes (IM) are mediated by mitofusins (MFN1 and MFN2) and optic atrophy 1 (OPA1), respectively [1]. Mitochondrial fission is executed by dynamin-related protein 1(Drp1) which is recruited from the cytosol to mitochondria by mitochondrial outer membrane protein Fis1, Mff4, Mid49, Mid51 [2]. OPA1, a dynamin‐like GTPase which is processed by two mitochondrial proteases OMA1 and Yme1L after entering into mitochondria, plays a role in remodeling cristae and the release of cytochrome c during apoptosis in addition to mediating mitochondrial inner membrane fusion [3–6]. Defective mitochondrial dynamic leads to mitochondrial dysfunction and are relevant with neurodegenerative disease [7–9]. Mitochondrial dysfunction will trigger an integrated stress response that endures periods of stress. Under stress, mitochondrial protease OMA1 will cleavage DELE1 into a short form that translocates from mitochondria to cytosol, where it binds to and activates the protein kinase HRI that phosphorylates eIF2α. Phosphorylated eIF2α enhances the expression of ATF4 which promotes translation of a wide range of adaptive genes [10–12].

In recent years, mutations at two mitochondrial homologous proteins CHCHD2 and CHCHD10 have been identified as a wild spectrum of neurodegeneration disease. Mutations of CHCHD2 are mainly linked to Parkinson’s disease [13]. Mutations of CHCHD10 are associated with ALS- and FTD-like symptoms [14–18], Charcot–Marie–Tooth disease type 2 (CMT2) [19, 20], spinal motor neuronopathy [21], motor neuron disease [15], and mitochondrial myopathy [22]. CHCHD2 and CHCHD10 belong to the mitochondrial coiled-coil-helix-coiled-coil-helix (CHCH) domain protein family whose members are nucleus-encoded mitochondrial small proteins containing twin CX9C motifs ((CX9C)2) characterized by two cysteine residues separated by nine amino acids and are imported to the mitochondrial intermembrane space [23].

CHCHD2 and CHCHD10 form a ∼220 kDa complex in the mitochondrial intermembrane space and cooperate to regulate mitochondrial function [24–26]. CHCHD2 has been reported to regulate oxidative phosphorylation (OXPHOS), promoting the expression of the COX4I2 subunit during stress [27, 28], inhibiting apoptosis [29]. Recently, it has been reported that CHCHD2 KO mice exhibited p62 inclusion formation and dopaminergic neuronal loss in an age-dependent manner [30].

CHCHD10 also regulates mitochondrial COX activity and mitochondrial respiration at hypoxia [31]. Many studies reported that CHCHD2 and CHCHD10 could play a role in mitochondrial dynamics and cristae organization. Disease-associated mutation of CHCHD2 and CHCHD10 has been found to lead to defects of mitochondrial dynamics and cristae.

At first, CHCHD2 and CHCHD10 were believed to be part of the mitochondrial contact site and cristae organizing system (MICOS) complex, which is crucial for mitochondrial membrane architecture and cristae organization, because they were observed enriched at cristae junctions, and their neurodegeneration associated mutations led to cristae abnormalities [14, 32–34]. However, the recent study would debate this view and suggest that neurodegenerative-disease-linked mutation of CHCHD10 or loss of CHCHD2 and CHCHD10 led to cristae abnormalities because of excessive processing of OPA1, while CHCHD2 or CHCHD10 single knockout did not lead to while CHCHD2 or CHCHD10 single knockout did not induce such effect [35, 36]. In MEF cells, both mutation CHCHD10-S59L and simultaneous loss of CHCHD2 and CHCHD10 were also found to cause mitochondrial integrated stress response (mtISR) [35, 36], however, the mechanism is unknown. In this study, we have investigated the mechanism that CHCHD2 and CHCHD10 regulate mitochondrial dynamics and mtISR. We show that a single loss of CHCHD2 could result in OPA1 processing and abnormalities of cristae. We propose that CHCHD2 and CHCHD10 bind OMA1 to inhibit its activity under normal conditions. We also observe that CHCHD2 and CHCHD10 translocate from mitochondria to cytosol resulting in the interaction with eIF2α, hence inhibiting eIF2α phosphorylation and mtISR under stress conditions.

### CHCHD2 and CHCHD10 regulate mitochondrial dynamics and ultrastructure

**Fig. 1:**
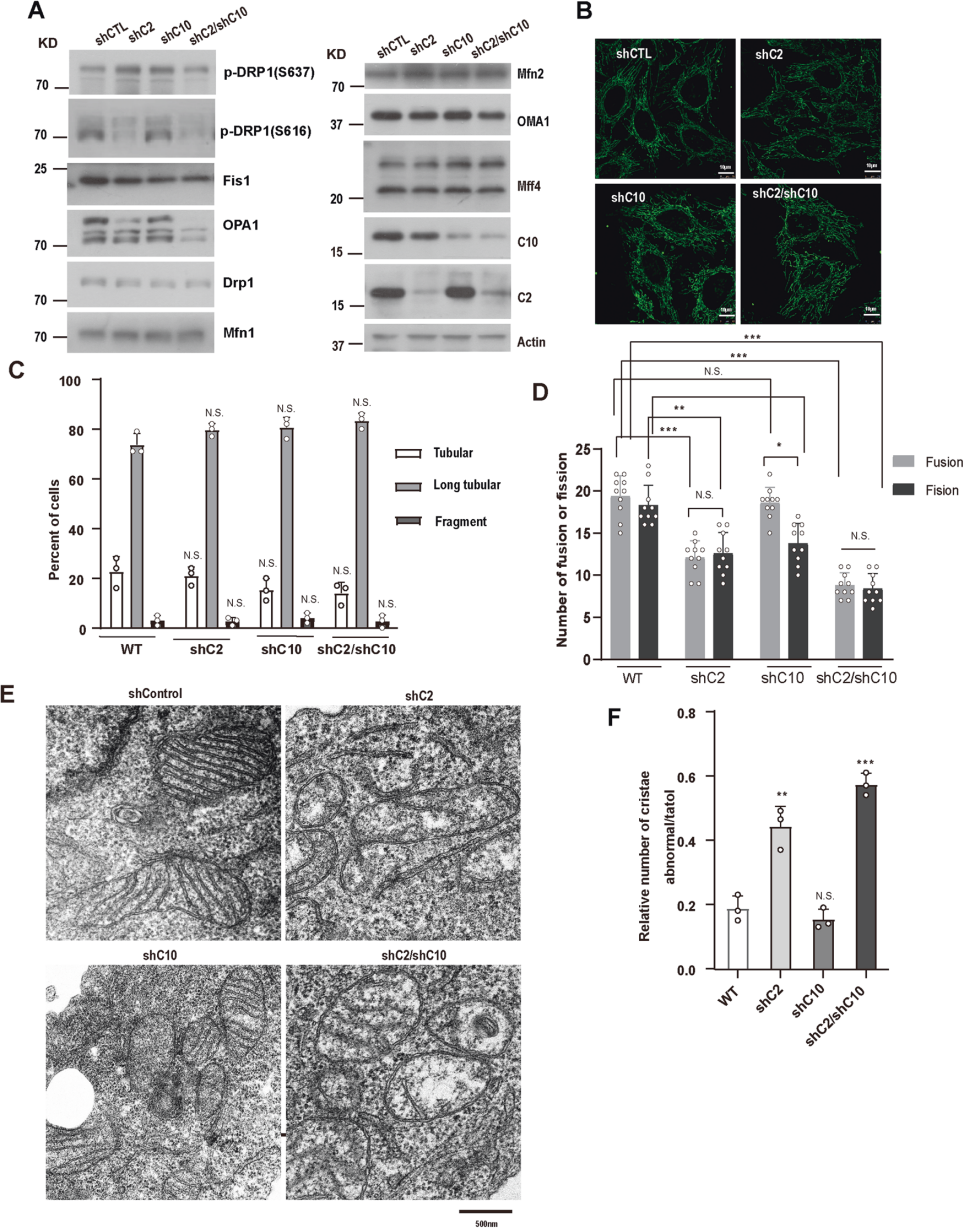
CHCHD2/CHCHD10 knockdown leads to a dynamic change of mitochondrial morphology and cristae abnormalities. **A** Whole-cell lysates of control, shCHCHD2, shCHCHD10, shCHCHD2/CHCHD10 HeLa cells were analyzed for indicated protein expression by immunoblot. β-Actin was used as a loading control. **B** Mitochondrial morphology in control, CHCHD2 knockdown, CHCHD10 knockdown, and CHCHD2/CHCHD10 knockdown HeLa cells immunostained for Hsp60 (Green) was visualized by confocal microscope. **C** Mitochondrial morphology described in **B** was counted according to the criteria detailed in Experimental Procedures (mean ± s.d. of *n* = 3 independent biological samples; two-way ANOVA with Tukey’s multiple comparisons correction, N.S. not significant). **D** Comparison of mitochondrial fusion and fission between control and shCHCHD2, shCHCHD10, shCHCHD2/CHCHD10 HeLa cells. Ten photoactivated mitochondria labeled with mito-PA-GFP were tracked by time-lapse microscopy for 20 min, and the number of mitochondrial fission and fusion events within 20 min was counted. Three independent cells were analyzed, the number of mitochondrial fission and mitochondrial fusions within 20 min were counted. Bars represent means ± S.D. of three independent experiments. Statistical significance analysis was used by two-way ANOVA with Sidak’s multiple comparisons test, *P < 0.05, **P < 0.01, ***P < 0.001, N.S. not significant. **E** Mitochondrial ultrastructure in control, shCHCHD2, shCHCHD10, shCHCHD2/CHCHD10 HeLa cells was analyzed by transmission electron microscope (TEM). **F** The relative number of mitochondria with abnormal cristae (ratio of abnormal mitochondria to total mitochondria) in control, shCHCHD2, shCHCHD10, shCHCHD2/CHCHD10 was counted. 100 mitochondria were measured from 20 cells. Statistical significance was assessed by one-way ANOVA with Dunnett’s multiple comparisons test; error bars represent means ± SD of three independent experiments; *P < 0.05,**P < 0.01,***P < 0.001, N.S., not significant.

To validate whether CHCHD2 and CHCHD10 have the function of maintaining mitochondrial morphology in human cells, we knocked down CHCHD2 or/and CHCHD10 in Hela cells by shRNA (Fig. 1A). We found that CHCHD2 or/and CHCHD10 knockdown cells showed similar mitochondrial morphology with the control cells (Fig. 1B, C). However, when we further assessed the activities of mitochondrial fusion and fission via a photoactivatable GFP (PA-GFP) assay [4, 37], we found that the rate of mitochondrial fusion and fission was significantly attenuated in CHCHD2 single knockdown as well as CHCHD2 and CHCHD10 double knockdown cells compared with control cells (Fig. 1D and Supplementary Movie 1–4). Notably, the alteration of slow mitochondrial fusion and fission was not due to the change of cell viability after CHCHD2/10 knockdown (Supplementary Fig. 1). To study the molecular mechanism of alternations in mitochondrial dynamics upon the loss of CHCHD2 and CHCHD10, we detected expression levels of mitochondrial proteins involved in mitochondrial fusion–fission process by Western blot. In CHCHD2 single knockdown Hela cells, long forms of OPA1 (L-OPA1) decreased significantly while short forms of OPA1 (S-OPA1) processed by OMA1 increased in comparison with control cells (Fig. 1A). Interestingly, CHCHD10 single knockdown did not alter the processing of OPA1, however, CHCHD2 and CHCHD10 double knockdown further increased the cleavage of L-OPA1. Fis1 and phosphorylated DRP1 (Ser616), both of which promote mitochondrial fission, were dramatically reduced in CHCHD2 knockdown and CHCHD2/CHCHD10 double knockdown cells. Other mitochondrial dynamics-related proteins, including Mfn1, Mfn2 and Mff had very similar expression levels between control cells and CHCHD2/CHCHD10 knockdown cells (Fig. 1A). Hence, these results demonstrated that despite no effect on mitochondrial morphology, loss of CHCHD2/CHCHD10 slowed down the rate of mitochondrial fusion and fission.

Since OPA1 also plays a role in mitochondrial cristae organization [38], reduction of L-OPA1 in response to CHCHD2/CHCHD10 knockdown also lead us to investigate whether CHCHD2 and CHCHD10 regulated mitochondrial ultrastructure in Hela cells. Utilizing the transmission electron microscopy (TEM), we observed that CHCHD10 single knockdown cell lines showed normal cristae structure while CHCHD2 single knockdown cells, as well as CHCHD2 and CHCHD10 double knockdown cells, exhibited cristae abnormalities (Fig. 1E, F). This result was consistent with the OPA1 processing resulting from CHCHD2 knockdown (Fig. 1A). Given CHCHD2 and CHCHD10 knockdown interrupted mitochondrial integrity, it prompted us to further investigate how such CHCHD2/10 deficiency-altered mitochondrial dynamics and ultrastructure would affect mitochondrial function and ROS production. To do this, we assessed cellular respiration by oximetry. The basal and maximal oxygen consumption was decreased in CHCHD2 or/and CHCHD10 knockdown cells when compared with wild-type control cells (Supplementary Fig. 2A). Mitochondrial membrane potential measured by Tetramethylrhodamine methyl ester (TMRM) fluorescence was also reduced in comparison with wild-type control cells (Supplementary Fig. 2B). The ATP production was decreased in CHCHD2 and CHCHD10 knockdown cells related to wild-type control cells (Supplementary Fig. 2C). In addition, mitochondrial ROS production measured by the reagent MitoSOX increased significantly in Hela cells after CHCHD2 and CHCHD10 knockdown (Supplementary Fig. 2D). Together, our results demonstrate that CHCHD2/CHCHD10 deficiency disrupts mitochondrial integrity, increases ROS production, and further compromises mitochondrial function in Hela cells.

### Loss of CHCHD2 and CHCHD10 induces mitochondrial ISR

**Fig. 2:**
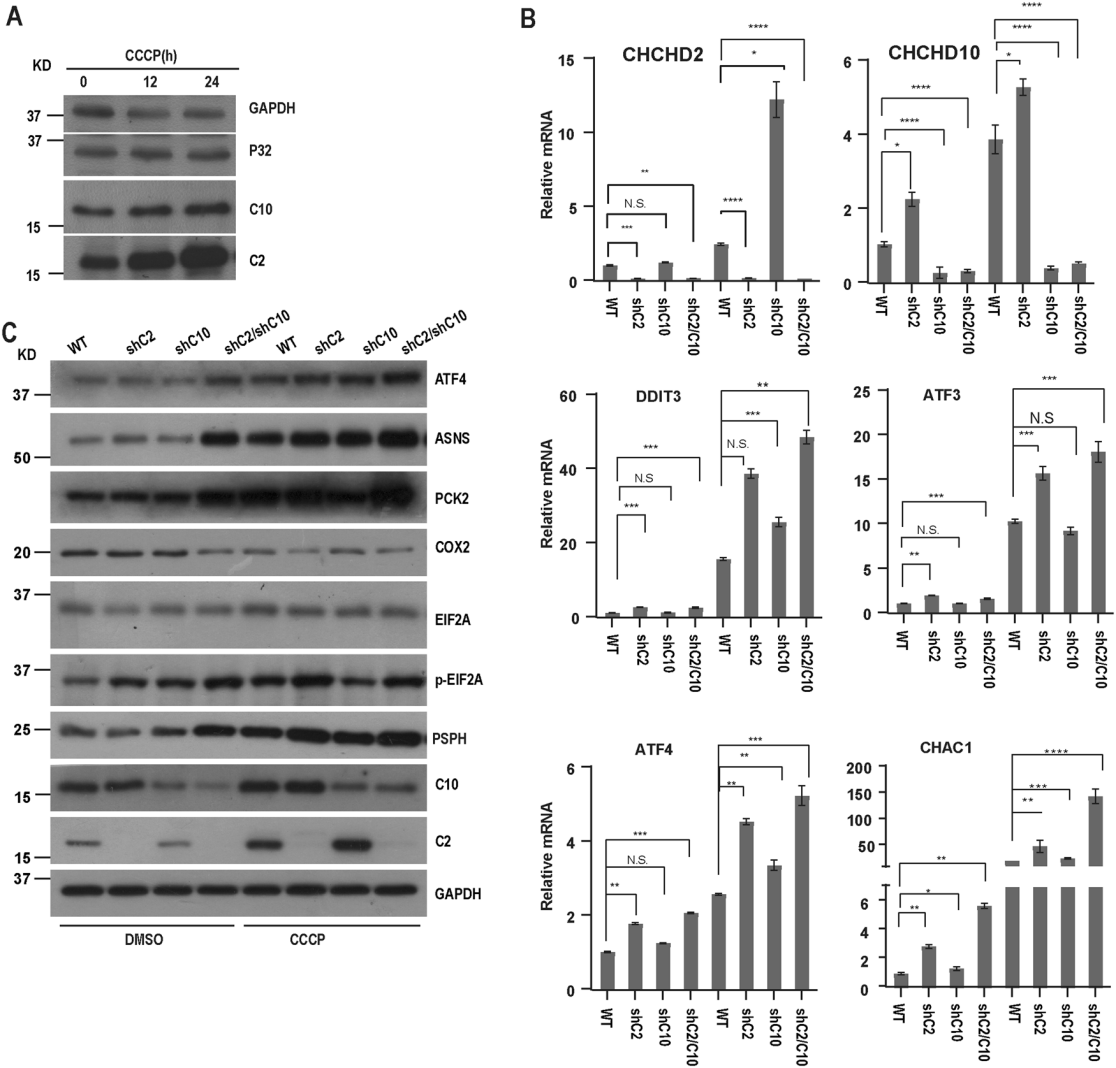
Loss of CHCHD2 and CHCHD10 promotes mitochondrial integrated stress response. **A** Whole-cell lysates of HeLa cells treated with DMSO or CCCP (10 mM, 12 h, or 24 h) respectively were analyzed for indicated protein expression by immunoblot. GAPDH was used as a loading control. **B** HeLa cells were infected with control, shCHCHD2, shCHCHD10, shCHCHD2/CHCHD10 lentiviral particles respectively and cultured for 7 days, treated with CCCP (10 mM) for 24 h, and then relative transcript levels of the indicated gene was analyzed by RT-QPCR. Statistical significance analysis was used by two-way ANOVA with Sidak’s multiple comparisons test, *P < 0.05, **P < 0.01, ***P < 0.001, N.S. not significant. **C** HeLa cells were infected with control, shCHCHD2, shCHCHD10, shCHCHD2/CHCHD10 lentiviral particles respectively and cultured for 7 days, treated with CCCP (10 mM) for 24 h, and then the indicated protein expression was analyzed by immunoblot. GAPDH was used as a loading control.

It has been previously reported that the abundance of CHCHD2 and CHCHD10 is increased after the loss of membrane potential [39]. To verify the report, we treated cells with carbonyl cyanide m-chlorophenylhydrazone (CCCP) and then detected CHCHD2 and CHCHD10 by Western blot assay. The results showd that CHCHD2 and CHCHD10 were robustly increased after CCCP treatment (Fig. 2A).

It has been also reported that CHCHD2 and CHCHD10 double knockout resulted in mitochondrial integrated stress response (ISR) instead of CHCHD2 or CHCHD10 single knockout in mouse embryonic fibroblasts at normal conditions [40]. CCCP treatment also triggers mitochondrial integrated stress response (ISR) [12]. To explore whether CHCHD2 and CHCHD10 can regulate ISR in human cells under normal conditions and stress conditions, we treated CHCHD2 or/and CHCHD10 knockdown cells with CCCP for 24 h. Then, we determined the mRNA (ATF3, ATF4, DDIT3, CHAC1) and proteins (ASNS, PCK2, PSPH, p-eIF2α) level of the ISR associated genes by RT-qPCR and Western blotting respectively (Fig. 2B, C). The ISR-related pathway proteins including ASNS, PCK2, and p-eIF2α and the mRNA1 levels of ATF3, ATF4, DDIT3, and CHAC1 were slightly increased after CHCHD2 or CHCHD10 single knockdown, while the levels of proteins and mRNAs of these genes were increased significantly after CHCHD2 and CHCHD10 double knockdown under normal conditions (Fig. 2B, C), which was consistent with what has been reported in the mouse [41]. However, following CCCP treatment, CHCHD2 single knockdown could increase mRNA and protein levels of genes associated with the ISR pathway (Fig. 2B, C). Together, our results indicate that CHCHD2 and CHCHD10 double knockdown induce ISR under normal conditions, while CHCHD2 single knockdown could promote ISR under stress conditions.

### CHCHD2 and CHCHD10 interact with OMA1 to inhibit its protease activity

**Fig. 3:**
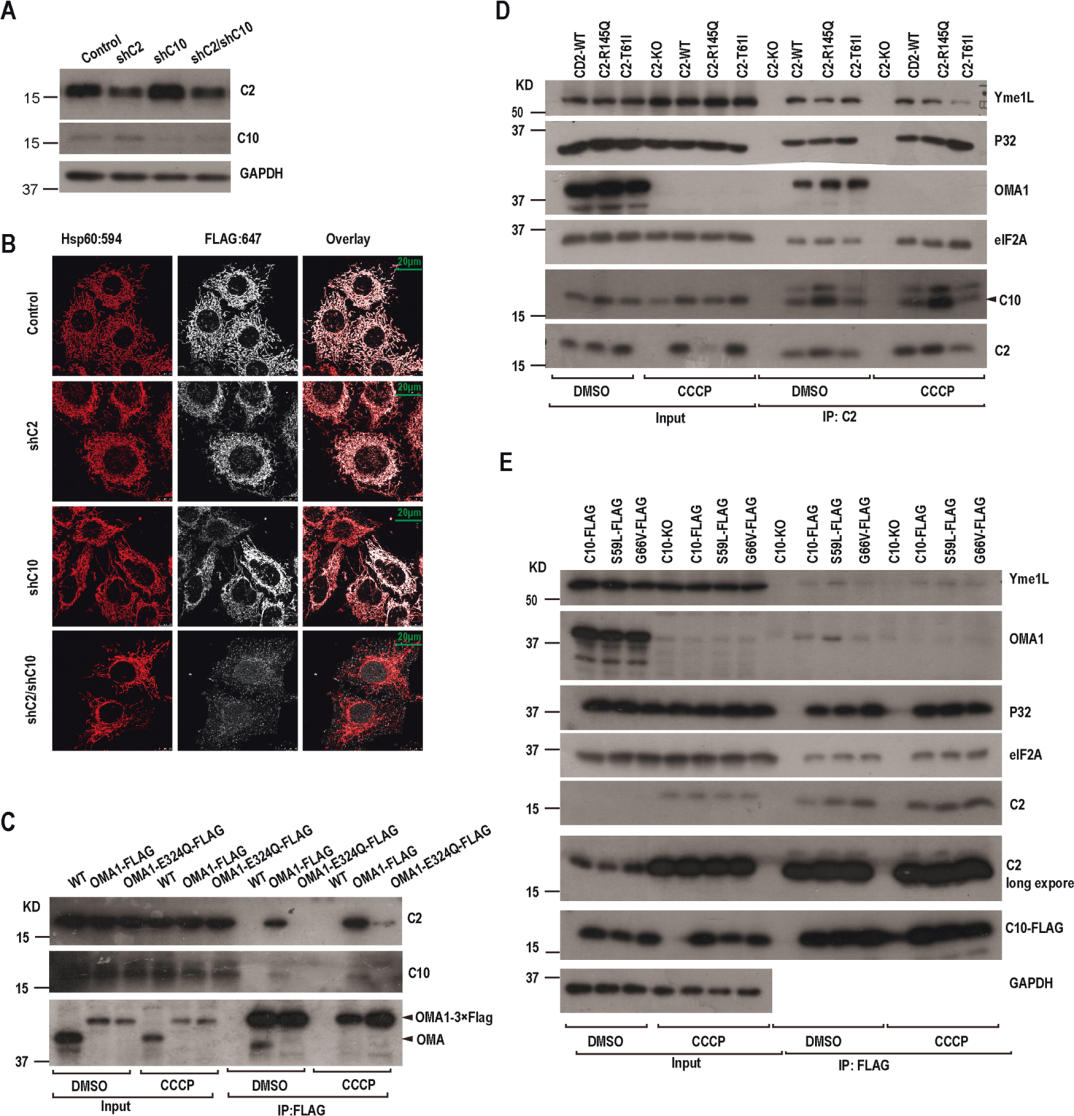
CHCHD2 and CHCHD10 interact with OMA1. **A** DELE1-FLAG-expressing HeLa cells were infected with control or shCHCHD2, CHCHD10, CHCHD2/CHCHD10 lentiviral particles and cultured for 5 days and then were treated with 10uM CCCP for 2 h. Cell lysates were subjected to immunoblot using the indicated antibodies. **B** DELE1-FLAG-expressing HeLa cells were infected with control or shCHCHD2, CHCHD10, CHCHD2/CHCHD10 lentiviral particles and cultured for 5 days and were immunostained with Hsp60, FLAG, and localization of DELE1-FLAG (white) was visualized by confocal microscopy. **C** HCT116 and OMA1 KO HCT116 cells expressing FLAG-tagged Mus OMA1-WT or OMA1-E324Q were treated with or without CCCP for 1 h. Then cells were lysed and immunoprecipitated with anti-OMA1 antibody, and the protein samples were subjected to immunoblot using the indicated antibodies. **D** CHCHD2 KO Hela cells expressing either exogenous wild-type CHCHD2 or its mutation T61I or R145Q were treated with or without CCCP for 8 h. Then cells were lysed and immunoprecipitated with anti-CHCHD2, and the protein samples were subjected to immunoblot using the indicated antibodies. **E** CHCHD10 KO Hela cells expressing exogenous FLAG-tagged CHCHD10 or its mutation S59L or G66V were treated with or without CCCP for 8 h. Then Cells were lysed and were immunoprecipitated with FLAG M2 resin, and the protein samples were subjected to immunoblot using the indicated antibodies.

As we knew, upon mitochondrial stress, DELE1 is cleaved by OMA1 in mitochondria and enters into the cytosol to interact with and assist HRI to phosphorylate the translation factor, eIF2α [10, 11]. To explore whether CHCHD2 and CHCHD10 regulated the cleavage of DELE1, we generated a Hela cell line stably expressing FLAG-tagged DELE1 by a retrovirus. Utilizing the immunofluorescence staining, we observed that CHCHD2 and CHCHD10 double knockdown promoted DELE1-FLAG to be accumulated into cytosol (Fig. 3A, B). These findings indicate that loss of CHCHD2 and CHCHD10 promotes the cleavage of DELE1 and triggers OMA1/DELE1-mediated ISR. We next investigate whether CHCHD10 and CHCHD2 would have a direct physical association with DELE1 to therefore suppress DELE1 cleavage. We, therefore, performed co-immunoprecipitation to examine whether DELE1 interacted with CHCHD2 and CHCHD10, and results showed that CHCHD10 and CHCHD2 failed to be precipitated by DELE1-FLAG under the normal conditions as well as following CCCP treatment, suggesting that CHCHD2 and CHCHD10 do not directly interact with DELE1 under both normal and stress conditions (Fig. 3C).

We also assessed whether OMA1 interacted with CHCHD2 and CHCHD10 by co-immunoprecipitation. We re-expressed OMA1-WT and its protease-dead mutation E324Q in OMA1 KO Hct116 cells. CHCHD2 and CHCHD10 were precipitated by OMA1-FLAG and OMA1-E324Q-FLAG under normal conditions and CCCP treatment. CCCP increased the interaction OMA1 with CHCHD2 and CHCHD10. To our surprise, OMA1-WT interacted with CHCHD2 more strongly than its mutant OMA1-E324Q (Fig. 3D). We speculated that CHCHD2 and CHCHD10 could interact with the active site of the protease of OMA1 to suppress its protease activity.

To further validate that CHCHD2 and CHCHD10 interact with OMA1. We re-expressed CHCHD2 WT, and its PD-related mutants CHCHD2-T61I, CHCHD2-R145Q in CHCHD2 knockout cells respectively, and re-expressed FLAG-tagged CHCHD10 WT and ALS-related mutants CHCHD10-S59L and CHCHD10-G66V in CHCHD10 knockout cells respectively. In parallel, we treated those cells with CCCP for 12 h. We performed co-immunoprecipitation experiments with CHCHD2 and FLAG antibodies respectively, followed by Western blot analysis. As reported previously, CHCHD2 and CHCHD10 interacted with P32, and CHCHD2 as well as CHCHD10 also interacted with the mitochondrial proteases OMA1 and YME1L (Fig. 3E, F). Since it has been reported that OMA1 is not required for basal turnover of CHCHD2 and CHCHD10 [41, 42] and loss of CHCHD2 and CHCHD10 lead to OPA1 processing and cleavage of DELE1, we hypothesized that CHCHD2 and CHCHD10 could regulate proteinase activity of Yme1L and OMA1 by binding them. Additionally, eIF2α, a cytoplasmic matrix protein, was also identified to interact with CHCHD2 and CHCHD10 and their mutations as well (Fig. 3E, F). Furthermore, CCCP promoted the interaction between eIF2α and CHCHD2/CHCHD10 (Fig. 3E, F).

### CHCHD2 and CHCHD10 translocate from mitochondria to the cytosol

**Fig. 4:**
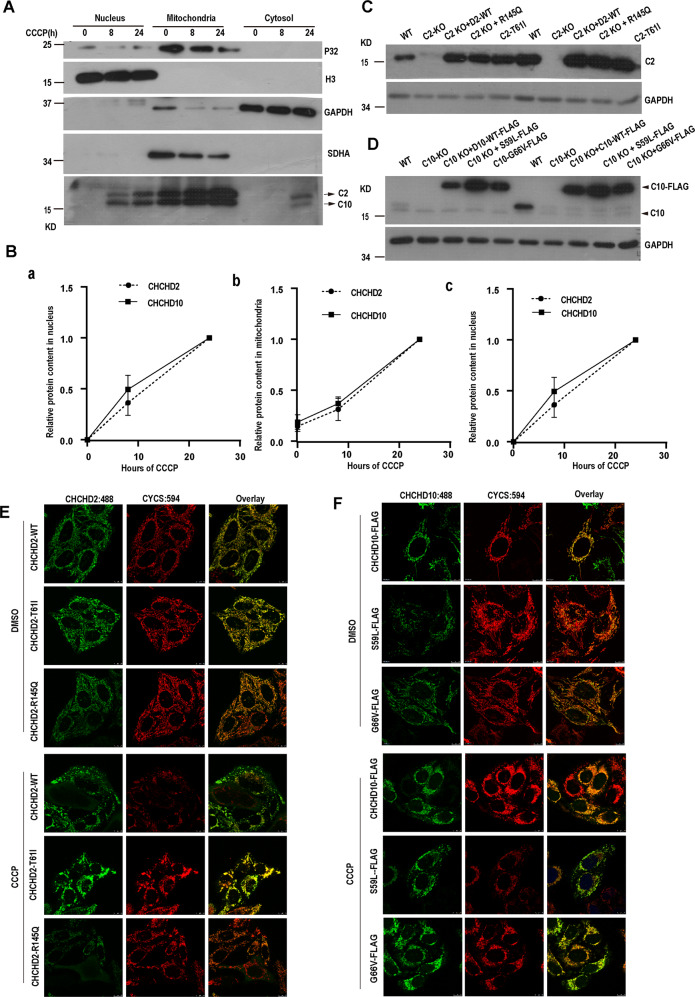
CCCP treatment increase CHCHD2 and CHCHD10 in mitochondria and cytosol. **A** Subcellular fractionation of HeLa cells treated with DMSO or CCCP (10 mM, 8 h or 24 h) respectively were analyzed for indicated protein expression by immunoblot. **B** Quantitative analysis of CHCHD2 and CHCHD10 in nucleus (a), in mitochondria (b) and in cytosol (c) from **A**. The levels of CHCHD2 and CHCHD10 in nucleus, mitochondria, and cytosol are normalized to those of H3, SDHA, and GAPDH respectively. *n* = 3 independent samples, data are presented as mean values ± SD. **C** Wild-type (WT) and clonal CHCHD2-knockout cells were transfected with indicated cDNA, treated with CCCP for 24 h and assayed by immunoblotting. **D** Wild-type (WT) and clonal CHCHD10-knockout cells, were transfected with indicated cDNA, and then treated with CCCP for 24 h and assayed by immunoblotting. **E** Subcellular localization of CHCHD2-WT, CHCHD2-T61I, or CHCHD2-R145Q in HeLa cells with or without CCCP treatment was analyzed by confocal microscopy. Mitochondria were visualized with CYCS staining. **F** Subcellular localization of FLAG-tagged CHCHD10 WT, CHCHD10-S59L, or CHCHD10-G66V in Hela Cells with or without CCCP treatment was analyzed by confocal microscopy. Mitochondria were visualized with Hsp60 staining.

It has been reported that CHCHD2 and CHCHD10 are mitochondrial proteins. Given that CHCHD2 and CHCHD10 interact with eIF2α which is a cytoplasmic protein, we hypothesized that CHCHD2 and CHCHD10 may localize in the cytosol as well. To validate this hypothesis, we isolated mitochondria, nucleus, and cytosol of Hela cells with or without CCCP treatment, followed by Western blot analysis. Results showed that in the absence of CCCP, CHCHD2 and CHCHD10 mainly co-fractionated with mitochondrial protein (Fig. 4A). Interestingly, by increasing the time period of CCCP treatment, an abundance of CHCHD2 and CHCHD10 was increased in the nuclear, mitochondria, and cytosol (Fig. 4A, B). To further assess whether CHCHD2 and CHCHD10 are translocated in cytosol under stress conditions, we expressed CHCHD2 and its PD-associated mutants CHCHD2-T61I and CHCHD2-R145Q in CHCHD2 KO Hela cells respectively, as well as expressed CHCHD10-FLAG, CHCHD10-S59L-FLAG, and CHCHD10-G66V-FLAG in CHCHD10 KO Hela cells respectively, followed by treatment with or without CCCP (Fig. 4C, D). Immunofluorescence analysis revealed that signals for CHCHD2 and CHCHD10 were mainly colocalized with CYCS marking mitochondria in the absence of CCCP, suggesting their mitochondria localization at normal conditions. Signals for CHCHD10-S59L showed aggregation at mitochondria, consistent with previous report under normal conditions [33, 40]. After 24 h of CCCP treatment, signals intensity for CHCHD2 and CHCHD10 and their mutations were enhanced and showed a diffuse pattern. Of special note, signals for CYCS marking mitochondria were disappeared in some cells because of mitophagy, however, signals intensity for CHCHD2, and CHCHD2-T61I and CHCHD2-R145Q, CHCHD10, CHCHD10-S59L-FLAG, CHCHD10-G66V-FLAG were increased at cytosol (Fig. 4E, F). Together, these results indicate that CHCHD2 and CHCHD10 are mainly localized at mitochondria at normal conditions, CCCP force translocation of CHCHD2 and CHCHD10 from mitochondria to cytosol and nucleus. Since simultaneous loss of CHCHD2 and CHCHD10 promotes phosphorylation of eIF2α under normal conditions and single loss of CHCHD2 promotes phosphorylation of eIF2α under CCCP treatment (Fig. 2C) and interaction of CHCHD2 and CHCHD10 with eIF2α is also increased after CCCP treatment (Fig. 3B, C). Thus, we speculated that CHCHD2 and CHCHD10 may suppress phosphorylation of eIF2α by binding it under CCCP treatment. Hence, CHCHD2 and CHCHD10 regulate ISR by inhibiting protease activity of OMA under normal conditions and phosphorylation of eIF2a under CCCP treatment.

### P32 regulates mitochondrial morphology and mtISR

**Fig. 5:**
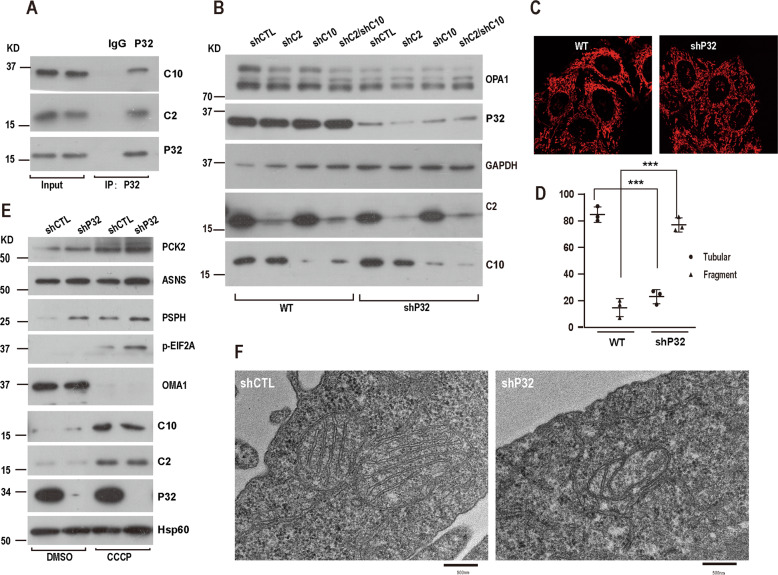
P32 interacts with CHCHD2 and CHCHD10 to regulate mitochondrial morphology and mtISR. **A** Hela cell lysates were immunoprecipitated with anti-P32 antibody or IgG, followed by immunoblotting using the indicated antibodies. **B** Lysates of P32-knockdown-Hela cells treated with DMSO or CCCP (10 uM, 124 h) analyzed for indicated protein expression by immunoblot. **C** Representative immunofluorescence images of mitochondrial morphology in control, shP32 Hela cells (mean ± s.d. of *n* = 3 independent biological samples; two-way ANOVA with Tukey’s multiple comparisons correction, ***P < 0.001). **D** Mitochondrial morphology described in **C** was counted according to the criteria detailed in Experimental Procedures. **E** Representative western blots of the indicated proteins in WT and shP32 Hela cells after 10uM CCCP treatment for 24 h. **F** Representative electron microscopic images and quantification of mitochondrial ultrastructure in WT or shP32 Hela cells.

P32, a multifunctional chaperone protein predominantly localizing in mitochondria, is functionally important for the maintenance of mitochondrial function. However, the function of P32 in mitochondrial morphology has been debated [43–46]. Given that P32 interacts with CHCHD2 and CHCHD10 (Fig. 3C–E, Fig. 5A), we were also interested to validate whether P32 regulates mitochondrial morphology and ISR. To this end, we generated P32 KD, CHCHD2/P32 DKD, CHCHD10/P32 DKD, and CHCHD2/CHCHD10/P32 triple KD (TKD) HeLa cells by lentivirus-mediated shRNA and checked the change of protein level of the mitochondrial dynamics related key proteins. The loss of P32 in Hela cells significantly led to a decrease of L-OPA1 and an increase of the S-OPA1 (Fig. 5B). Immunofluorescence analysis revealed that P32 knockdown led to remarkable mitochondrial fragmentation in about 70% of Hela cells, whereas wild-type (WT) Hela cells showed almost all tubular mitochondria (Fig. 5C, D).

To test whether P32 could regulate ISR, we treated WT or shP32 Hela cells with CCCP for 12 h. P32 knockdown resulted in an increase of ISR proteins under both normal conditions and CCCP treatment (Fig. 5E), suggesting that loss of P32 promotes ISR under normal conditions and stress conditions. Consistent with OPA1 processing, P32 knockdown also resulted in mitochondrial cristae abnormalities (Fig. 5E). Together, these data suggest that P32 coordinates with CHCHD2 and CHCHD10 to regulate mitochondrial morphology and ISR.

### OMA1 and Yme1L are responsible for the degradation of CHCHD2 and CHCHD10

**Fig. 6:**
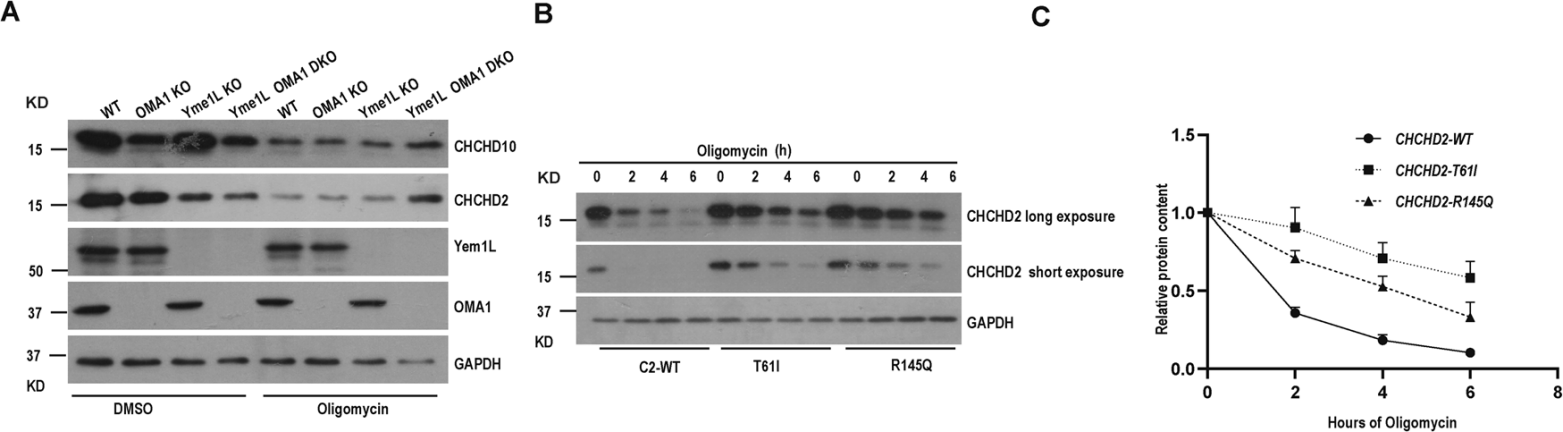
Yme1L and OMA1 are responsible for degrading CHCHD2 and CHCHD10. **A** Immunoblot of OMA1 KO, Yme1L KO, OMA1 Yme1L DKO, and WT HeLa cells treated with dimethyl sulfoxide (DMSO) or 10 μm Oligomycin for 8 h. GAPDH served as loading controls. **B** Immunoblot of WT and CHCHD2 KO HeLa cell lines which stably express CHCHD2 WT, T61I or R145Q mutation where indicated, treated with either vehicle or 125 μm Oligomycin for 6 h. GAPDH served as the loading control. **C** Quantification of levels CHCHD2 and its mutation CHCHD2-T61I and CHCHD2-R145Q from **B.** The relative protein levels were evaluated by densitometry analysis using ImageJ software and were quantified for the ratio of CHCHD2/GAPDH, T61I /GAPDH, and R145Q/GAPDH (*n* = 3 independent experiments). The data are presented as mean ± SD.

Although previous studies reported that CHCHD2 and CHCHD10 could be degraded by OMA1, when cells are subject to mitochondrial stressor, Actinoin, OMA1 knockout could not entirely restore CHDHD2 and CHCHD10 degradation after Actinonin treatment, suggesting that, besides OMA1, there are other proteases responsible for degrading CHCHD2 and CHCHD10 [41]. Given mitochondrial protease OMA1 and Yme1L interact with CHCHD2 and CHCHD10 (Fig. 3D, E), we assess whether Yme1L is responsible for the degradation of CHCHD2 and CHCHD10. We generated OMA1 and Yme1L double knockout Hela cell lines with CRISPR-Cas9 system, and treated the cell lines with Oligomycin, a mitochondrial complex V inhibitor. Western Blot analysis showed that Oligomycin treatment caused CHCHD2 and CHCHD10 degradation, however, OMA1 and Yme1L knockout almost completely inhibited the degradation of CHCHD2 and CHCHD10 following oligomycin treatment (Fig. 6A). These data indicate that Yme1L is also required for stress-induced degradation of CHCHD2 and CHCHD10.

Since CHCHD2 and CHCHD10 can be degraded under mitochondrial stress, suggesting that the degradation of CHCHD2 and CHCHD10 may play a critical role in the maintenance of cell function. We test whether the degradation rate of neurodegenerative disease-related CHCHD2 mutants would behave similarly to CHCHD2 in wildtype. To this end, we re-expressed wild-type CHCHD2 and their mutants T61I and R145Q in CHCHD2 knockout cells and then treated these cell lines with Oligomycin in a time-dependent manner. Western Blot analysis showed that the degradation rate of CHCH2 mutants, T61I and R145Q, was significantly slower than that of the wild-type CHCHD2 (Fig. 6B, C), indicating that neurodegenerative disease-related CHCHD2 mutants are resistant to degradation by Yme1L and OMA1 under mitochondrial stress.

## Discussion

CHCHD2 and CHCHD10 are homologous mitochondrial proteins and their mutations are identified with neurodegenerative disease [23]. Previous studies in mice have shown that CHCHD2 or CHCHD10 single knockout do not alter mitochondrial shape and ultrastructure, but CHCHD2 and CHCHD10 double knockout increase processing of and subsequent mitochondrial fragmentation and cristae abnormities [41].

In this study, we report that CHCHD2 single knockdown as well as CHCHD2 and CHCHD10 double knockdown can slow down mitochondrial fusion and fission (Fig. 1D). Because the rates of mitochondrial fusion and division are still similar, CHCHD2 or/and CHCHD10 knockdown cells show normal mitochondrial morphology. We found that, CHCHD2 knockdown, but not CHCHD10, caused OPA1 processing and disrupted mitochondrial cristae in HeLa cells, and CHCHD2 and CHCHD10 double knockdown was able to further such adverse effect (Fig. 1E, F).

Mitochondrial dysfunction could trigger mtISR. Simultaneous loss of CHCHD2 and CHCHD10 could leads to mtISR in mouse [35, 36]. Under normal conditions, CHCHD2 and CHCHD10 single knockdown does not activate ISR, while CHCHD2 and CHCHD10 double knockdown leads to mtISR (Fig. 2B, C), which is consistent with previous reports [41]. However, we discovered that CHCHD2 single knockdown promotes mtISR triggered by CCCP treatment (Fig. 2B, C). Moreover, CHCHD2 and CHCHD10 are significantly increased after CCCP treatment (Fig. 2A), so we speculate that under normal conditions, the protein content of CHCHD2 or CHCHD10 is sufficient to inhibit mtISR, while under stress conditions, mitochondria damaged, the amount of CHCHD2 and CHCHD10 is insufficient to inhibit mtISR.

OMA1 maintains low activity under normal physiological conditions but can be activated to process OPA1 to regulate mitochondrial dynamics and cleave DELE1 to activate mtISR under stress conditions [47]. There are three possible reasons why CHCHD2 and CHCHD10 knockdown leads to the processing of OPA1 and DELE1: loss of CHCHD2 and CHCHD10 induce bioenergetic stress that activates OMA1; CHCHD2 and CHCHD10 bind to OPA1 and DELE1 to inhibit its processing by protease; CHCHD2 and CHCHD10 bind OMA1 to suppress its activity. We observed that either CHCHD2 or CHCHD10 knockdown resulted in similar decrease of mitochondrial membrane potential and ATP production (Supplementary Fig. 2). However, CHCHD2 knockdown, not CHCHD10, increased OMA1-mediated OPA1 cleavage in Hela cells (Fig. 1A). These data indicate that the lack of CHCHD2 activates OMA1 not because of bioenergetic collapse.

CHCHD2 and CHCHD10 could bind OMA1 but not OPA1 and DELE1 under normal conditions (Fig. 3C, E, Supplementary Fig. 3), so we speculated that CHCHD2 and CHCHD10 inhibitted the activity of OMA1 by binding OMA1, but not by binding OPA1 and DELE1 to suppress their processing under normal conditions.

CHCHD2 single knockdown leads to activation of OMA1, while CHCHD10 single knockdown does not lead to OMA1 activation, and CHCHD2 and CHCHD10 double knockdown lead to further activation of OMA1. Therefore, we hypothesized that CHCHD2 played a major role and CHCHD10 played a minor role in repressing the activity of OMA1 under normal circumstances. By immunoprecipitation, we observed that CHCHD2 and CHCHD10 also interacted eIF2α under normal conditions, and surprisingly CHCHD2 and CHCHD10 interacted with eIF2α more strongly under stress conditions than under normal conditions (Fig. 3D–F). In addition, more CHCHD2 and CHCHD10 entered to the cytosol under stress conditions (Fig. 4), and loss of CHCHD2 and CHCHD10 increased the phosphorylation of eIF2α under normal conditions and stress conditions (Fig. 2B). Given that eIF2α is phosphorylated during ISR initiation, therefore, we hypothesized that CHCHD2 and CHCHD10 interactted with eIF2a to inhibit its phosphorylation, thereby regulating ISR under stress conditions.

**Fig. 7:**
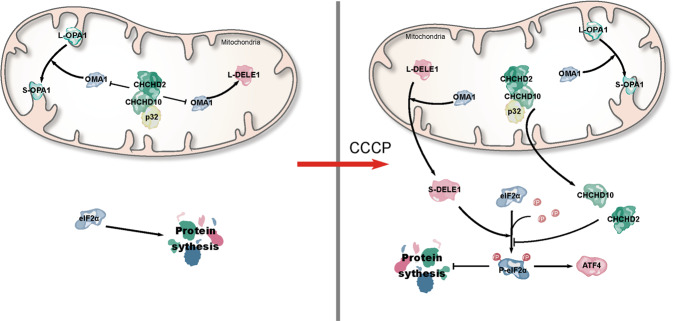
Model of CHCHD2 and CHCHD10 regulating OMA1 activity and mtISR. CHCHD2 and CHCHD10 mainly suppress the OMA1 activity and inhibit phosphorylation of eIF2A at normal conditions by binding them, and loss of CHCHD2 and CHCHD10 activates OMA1 to cleavage OPA1 and DELE1; at stress conditions (loss of membrane potential), CHCHD2 and CHCHD10 increase and translocate from mitochondria to cytosol to suppress phosphorylation of eIF2A to inhibit ISR.

Based on the above results, we propose a model: at normal conditions, CHCHD2 and CHCHD10 mainly suppress the OMA1 activity and inhibit phosphorylation of eIF2α by binding them, while loss of CHCHD2 and CHCHD10 activate OMA1 to cleavage OPA1 and DELE1; at stress conditions (loss of membrane potential), CHCHD2 and CHCHD10 increase and translocate from mitochondria to the cytosol to suppress phosphorylation of eIF2α to inhibit ISR (Fig. 7).

Finally, we discovered that P32, a partner of CHCHD2 and CHCHD10, can also regulate mitochondrial morphology and ISR. However, the detailed mechanisms are still to be investigated.

Mutations in CHCHD2 and CHCHD10 can lead to neurological diseases, while CHCHD2 and CHCHD10 are degraded by proteases under stress conditions. We find that Yme1l and OMA1 cooperate to degrade CHCHD2 and CHCHD10 under stress conditions (Fig. 6A), and the degradation rate of the PD-related mutations of CHCHD2, CHCHD2-T61, and CHCHD2-R145Q is significantly slower than that of the wild-type CHCHD2 (Fig. 6B).

## Materials and methods

### Cell culture

HeLa (CCL-2, ATCC), 293 T (CRL-3216, ATCC), HCT116 (CCL-247, ATCC), and GP2-293(631512, TaKaRa) cells were cultured in Dulbecco’s Modified Eagle Medium (DMEM) (11995065, Gibico) containing 4.5 g l − 1 glucose, L-Glumine and sodium pyruvate supplemented with 10% fetal bovine serum (ST30-3302, PAN, Germany), and 1% sodium pyruvate at 37 °C under 5% CO2 conditions. All cell lines were tested for absence of mycoplasma contamination and authenticated using the short tandem repeat (STR) method.

### Stable cell generation

Stable expression cell lines and rescued knockout cell lines were generated using retrovirus infections. Human CHCHD2 cDNA and its mutants were cloned into a modified pMSCV-puro (K1062-1, Clontech) vector whose puromycin resistance marker was deleted. Human DELE1 cDNA, CHCHD10 cDNA, CHCHD10-S59L, and CHCHD10-G66V cDNA with a C‐terminal 3 × Flag tag were also cloned into the modified pMSCV-puro vector whose puromycin resistance marker was deleted. Mouse OMA1 cDNA and OMA-E324Q were cloned into pMSCV‐puro vector with a C‐terminal 3 × Flag tag. Retroviral particles were generated by transient transfection of GP2-293 cells seeded in 6-cm cell culture plates with VSV-G (#8454, Addgene) and transfer plasmid using HighGene Transfection reagent (RM09014, ABclonal). Forty-eight hours later, the supernatant was collected and filtered, and then was added to cells. Cells were incubated with virus for 8 h with 10 μg/ml polybrene and then the medium was discarded and replaced with a fresh medium. One week after viral transduction, stable expression cell lines and rescued knockout cell lines were verified by Western Blot and immunoblotting.

CHCHD2 and CHCHD10 knockdown cell lines were generated using a modified retroviral vector as described previously [4]. P32 knockdown cell lines were generated using pLKO.1 puro (8453, Addgene). The following target sequences for gene knockdown were:

5′‐CAGTGGAGGAAGTAATGCT‐3′ for CHCHD2;

5′‐CCCTGAAGCAGTGCAAGTA‐3′ for CHCHD10;

5′‐GGATGAGGTTGGACAAGAAGA‐3′ P32.

Retrovirus particles for shCHCHD2 and shCHCHD10 were generated as above. Lentivirus particles for shP32 were generated by transient transfection of 293 T cells seeded in 6-cm cell culture plates with VSV-G (#8454, Addgene), pCMV-dR8.2 dvpr(#8455, Addgene), and lentiviral vector using HighGene Transfection reagent (RM09014, ABclonal), 48 h later, the supernatant was collected and filtered. Cells were incubated with virus for 8 h with 10 μg/ml polybrene and then the medium was discarded and replaced with fresh medium. Five days after infection, the loss of target protein expression was verified by immunoblotting.

Hela cells lacking CHCHD2 and CHCHD10, Hct116 cells lacking OMA1 or/and Yme1L were generated by CRISPR/Cas9 gene editing, as described previously [48]. The following guide sequences were used:

5′‐ACATTAGCATCCACCTCACG‐3′ for OMA1;

5′‐TGTCCAAGTGTTGGCCCCCG‐3′ for Yme1L;

5′‐CGGCCAGGTGAGACCATCGC-3′ for CHCHD2;

5′‐GAGATGGCGACCACGGCCGCA-3′ for CHCHD10.

Designed Oligos were cloned into LentiCRISPR-V1 plasmid (49535, Addgene) and then were packaged into lentivirus in 293 T cells as above. At 48 h post-transfection, the supernatant containing lentiviral particles was collected and used for infecting cells with 10 μg/ml polybrene (H9268, Sigma‐Aldrich). Eight hours later, the medium was discarded and replaced with a fresh medium. Twenty-four hours later, 2 μg/ml puromycin was added to the medium to select and establish stably transfected cells. Then, the single cells were sorted into 96‐well dishes for the screen of knockout lines. The surviving clones were expanded, selected, and analyzed by immunoblotting. The genomic region flanking the targeting sequence was amplified by PCR and subjected to DNA sequencing.

### Reagents and antibodies

Reagents used in this paper are listed as follows: Dimethyl sulfoxide (DMSO, 41639, Sigma-Aldrich); Carbonyl cyanide 3chlorophenylhydrazone (555-60-2, Sigma-Aldrich); Puromycin (A1113803, Invitrogen); Digitonin (D141, Sigma-Aldrich); Oligomycin (1404-19-9, Santa Cruz Biotechnology); and Opti‐MEM (Thermo Fisher Scientific).

The following commercial antibodies were used for Western Blot: anti‐Flag (Sigma‐Aldrich, F1804, 1:5000 dilution); anti‐OMA1, (Santa Cruz Biotechnology, H‐11, 1:200 dilution); anti‐Yme1L (Proteintech, 11510‐1‐AP, 1:3000 dilution); anti‐SDHA (Proteintech, 14865‐1‐AP, 1:3000 dilution); anti‐OPA1 (BD, 612607, 1:2000 dilution), Anti-Drp1 (BD, 611738, 1:2000 dilution); Anti-ASNS (14681-1-AP, Proteintech, 1:1000 dilution); Anti-PCK2(14892-1-AP, Proteintech, 1:1000 dilution); Anti-PSPH (Proteintech, 14513-1-AP, 1:1000 dilution); Anti-Fis1 (Proteintech, 10956-1-AP, 1:1000 dilution); Anti-Mff (Proteintech, 17090-1-AP, 1:1000 dilution); Anti-CHCHD2(Proteintech, 19424-1-AP, 1:1000 dilution); Anti-CHCHD10 (Proteintech, 25671-1-AP, 1:1000 dilution); Anti-P32 (Abclonal, A1883, 1:5000 dilution); Anti-eIF2α (Cell Signaling Technologies, #5324, 1:3000 dilution); Anti-Phosphorylated eIF2α(Cell Signaling Technologies, #3398, 1:1000 dilution), Anti-GAPDH (Cell Signaling Technologies, #5174, 1:10000 dilution); Anti-phosphorylated Drp1 (Ser616) (Cell Signaling Technologies, #3455, 1:1000 dilution); Anti-phosphorylated Drp1 (Ser637) (Cell Signaling Technologies, #4867, 1:1000 dilution); Anti-Actin (Sigma, A5441, 1:10000 dilution); Anti Hsp60 (Proteintech, 15282-1-AP, 1:5000 dilution); Anti-SDHA (Proteintech, 14865-1-AP, 1:5000 dilution).

### Immunostaining

Cells grown on coverslips were fixed for 20 min with 4% paraformaldehyde at first, then washed three times with PBS, and permeabilized for 10 min with 0.1% Triton X-100 in PBS. After three washes with PBS, cells were blocked with 10% FBS in PBS for 1 h at room temperature, then incubated with primary antibody for 1 h at room temperature. After three washes with PBS, cells were incubated with secondary antibodies for 1 h at room temperature. Finally, cells were washed three times with PBS and then were analyzed by using a Leica confocal microscope.

### Confocal microscopy and image processing

Fixed or living cells were visualized by confocal microscopy with a Leica Sp8 microscope with a 633 numerical aperture 1.35 oil objective [49]. To determine mitochondrial morphology, 100 cells were randomly selected for quantitative analysis and visually scored into three classifications (tubular, short tubular, fragmented). Tubular refers to cells with only tubular mitochondria. Tubular refers to cells in which greater than half of the mitochondrial mass existed as tubules as opposed to spherical fragments. Short tubular refers to cells in which less than half of the mitochondrial mass existed as tubules. In addition, all cells in this class contained at least three clearly tubular mitochondria. Finally, fragmented refers to cells that contain spherical mitochondrial fragments with no more than two short tubules found.

### Electron microscopy

The procedure for transmission electron microscopy (TEM) was performed according to the previous report [50]. The 100 mM sodium cacodylate buffer was replaced by 100 mM phosphate buffer without CaCl2. The sections were supported on copper grids and then post-stained in uranyl acetate for 10 min and then in lead citrate for 15 min, and the stained sections were imaged onto negatives using a JEM-1400 plus electron microscope operated at 100 kV.

### Quantitative real‐time PCR analysis

RNA from cells or tissues was extracted using RNAiso Plus reagent (TaKaRa Biotechnology, 9109) following the manufacturer’s protocol. And the cDNA was synthesized using the ABScript II cDNA First Strand Synthesis Kit (ABclonal, RK20400) according to the manufacturer’s instructions. Then, the cDNA samples were used as templates for quantitative real‐time quantitative PCR analysis by using an SYBR Green Select Master Mix (ABclonal, RK21203) and the iCycler real‐time PCR Detection System (Bio‐Rad). The fold change of target mRNA expression was calculated using the 2^−ΔΔCT^ method. The β‐ACTIN or GAPDH housekeeping gene was used for normalization. The primers used in this study were as followed:

CHCHD2: forward, 5′‐GTGGAGGAAGTAATGCTGAGCC‐3′, and reverse, 5′‐CACAGAGCTTGATGTCACCCTG‐3′, CHCHD10: forward, 5′‐ATCTGGTGTTGTGGTCTGGCTG‐3′, and reverse, 5′‐GTGAGTGAGTGGACCCCGAC‐3′, ATF3: forward, 5′‐GGAGCCTGGAGCAAAATGATG‐3′, and reverse,5′‐AGGGCGTCAGGTTAGCAAAA‐3′, CHAC1: forward, 5′‐GTGGTGACGCTCCTTGAAGA‐3′, and forward, 5′‐TTCAGGGCCTTGCTTACCTG‐3′, DDIT3: forward, 5′‐AGCCAAAATCAGAGCTGGAA‐3′, and forward, 5′‐TGGATCAGTCTGGAAAAGCA‐3′, ATF4: forward, 5′‐CAGCAAGGAGGATGCCTTCT‐3′, and forward, 5′‐CCAACAGGGCATCCAAGTC‐3′, β‐ACTIN: forward, 5′‐GGCATGGGTCAGAAGGATT‐3′, and reverse, 5ʹ‐CCACACGCAGCTCATTGTA‐3′.

### Subcellular fractionation

Cell fractionation and mitochondria isolation were performed as previously reported [51]. Briefly, following the 8 h or 24 h CCCP treatment, cells were collected and centrifuged at 400 × g for 5 min at 4 °C. The cell pellet was washed once with PBS and permeabilized with 10 mg/ml digitonin (Sigma, D141) in PBS for 10 min at room temperature. Cells were centrifuged at 10,000 × g for 10 min at 4 °C, and supernatants were collected to a fresh tube (cytosolic fraction). The pellets were resuspended in the mitochondrial lysate buffer (10 mM Tris-HCl pH 7.4, 150 mM NaCl, 2 mM EDTA, 0.2% Triton, 0.3% NP40, protease inhibitor cocktails) for 30 min on ice and centrifuged at 10,000 × g for 20 min at 4 °C. The supernatants and the pellets were the mitochondrial fraction and nuclear fraction respectively.

### Western blotting and co‐immunoprecipitations

Western blotting and co‐immunoprecipitation (co‐IP) analyses were performed as previously described. In brief, cells were lysed with RIPA buffer (Beyotime, P0013B) and complete protease inhibitor (Roche). The proteins were loaded onto an SDS–polyacrylamide gel, separated by electrophoresis, and blotted onto a PVDF membrane (Merck Millipore). For the co‐IP, all steps were performed at 4 °C, cells were solubilized with IP buffer (20 mM Tris-HCl pH = 7.4, 150 mM NaCl, 2 mM EDTA, 1% Triton X‐100 and protease inhibitor mixture) for 1 h. The lysates were centrifuged at 12,000 × *g* for 15 min and the supernatant was subsequently incubated with anti-CHCHD2 antibody and protein G conjugated magnetic beads (Bio-Rad, G-1614823) or anti‐Flag M2 affinity gel (Sigma‐Aldrich, A2220) at 4 °C overnight. The affinity gel was washed five times with lysis buffer, and then, the proteins were recovered by boiling the beads in sample buffer and analyzed by Western blotting.

### ROS measurement

ROS levels were measured using MitoSox (M36008, Thermo Fisher Scientific). In brief, cells were stained with 5 μM MitoSox at 37 °C for 30 min and then analyzed by Cyto FLEX LX (Beckman Coulter). CCCP was used as a control of the technique.

### Membrane potential measurement

Mitochondrial membrane potential (Δψm) was measured using TMRM(M20036, Thermo Fisher Scientific). In brief, cells were stained with 200 nM TMRM at 37 °C for 15 min and then analyzed by Cyto FLEX LX (Beckman Coulter). CCCP was used as a control of the technique.

### ATP measurement

Cellular ATP levels were measured using an ATP assay kit (Celltiter-Glo Luminescent Cell Viability Assay, G7573, Promega) according to the manufacturer’s instructions. Briefly, cells were planted in 96-well plates and cells were cultured in DMEM without glucose (11966025, Gibco) containing L-glutamine supplemented with 10 mM galactose (G5388, Sigma), 100 µM non-essential amino acids, 1 mM sodium pyruvate, and 10% FBS (ST30-3302, PAN). Whole-cell lysates were generated using the luciferase reporter lysis buffer. Luminescence was measured using the microplate reader and the values were normalized to the protein concentration. CCCP was used as a control of the technique.

### Cell proliferation assay

Cell proliferation was measured using a Cell Counting Kit (CCK-8, C0042, Beyontime) according to the manufacturer’s instructions. The absorbance of control and knockout cells was measured with the microplate reader at 450 nm.

### Seahorse

Oxygen consumption rates (OCR) of cells were measured with a Seahorse Extracellular Flux Analyzer XF96 (Agilent), according to the manufacturer’s instructions. In brief, Hela cells were seeded in an XF96‐well plate at a density of 1.5 × 10^4^ per well and allowed to attach overnight. The standard ‘mitochondria stress test’ was performed consisting of basal measurements followed by measurements after sequential addition of 1 μm oligomycin, 1.5 μm FCCP, and 1 μm of rotenone and antimycin. Data are presented as means ± standard error of the mean for five replicates. Protein concentrations determined by BCA assay were used to normalize the OCRs.

### Photoactivatable GFP assay

Mitochondrial targeted DesRed (mito-DsRed) and photoactivatable GFP (PA-GFP) targeted to the mitochondrial matrix (mito-PA-GFP) were stably expressed in cells and performed PA-GFP assay. Within a single cell, a small subset of mitochondria was photoactivated by excitation at 405 nm, and then the mitochondrial fusion and fission events were tracked by time-lapse microscopy for about 20 min.

### Statistical analyses

All statistical analyses were performed using Excel (Microsoft) or Prism (Graphpad 8.0). All statistical results are presented as the mean ± standard deviation (S.D.), and *p*-values were calculated using two-tailed Student’s *t*-test for pairwise comparisons, one-way ANOVA with Dunnett’s multiple comparisons test for multiple comparisons, and two-way ANOVA with Tukey’s multiple comparisons test for multiple comparisons test involving two independent variables. *p*-values < 0.05 were considered significant. In all experiments, variances between groups were not statistically different.

### Statistics and reproducibility

All experiments were performed independently at least three times unless stated otherwise in the figure legend. The sample size was chosen in accordance with the general standards and prior publications in the respective fields [37, 52]. No statistical method was used to predetermine the sample size. No data were excluded from the analyses. The experiments were not randomized. The investigators were not blinded to allocation during experiments and outcome assessment.

## Supplementary information

Reproducibility checklist

Video S1

Video S2

Video S3

Video S4

Supplementary materials

Supplementary Figure 1

Supplementary Figure 2

Supplementary Figure 3

## Data Availability

The data generated or analyzed are included in this article and its supplementary files.
