# Supplementary material for: CHCHD2 and CHCHD10 regulate mitochondrial dynamics and integrated stress response
Source: Cell Death Dis. 2022 Feb 16;13(2):156. doi: 10.1038/s41419-022-04602-5 (PMC8850591; doi:10.1038/s41419-022-04602-5)
Supplement: Supplementary file 6 — Supplementary materials [file 41419_2022_4602_MOESM6_ESM.docx]

**Supplementary Figure 1**

Hela cells were infected with pLKO or shCHCHD2, CHCHD10 lentivirus to generate stable knockdown cells.  The cells incubated with normal growth media for the indicated time and subjected to CCK-8 assay. % of cell viability plotted with SD as error bars. Student’s t test, *p < 0.05, **p < 0.01, N.S., not significant.

**Supplementary Figure 2**

**(A)** OCR of WT, CHCHD2 knockdown, CHCHD10 knockdown, CHCHD2 and CHCHD10 double knockdown Hela cells measured by the Seahorse instrument. Injections of oligomycin (Oligo), carbonyl cyanide-4-(trifluoromethoxy)phenylhydrazone (FCCP) and rotenone (Rot)/antimycin (Ant) are indicated. Plot is representative of at least 5 biological replicates performed on at least two occasions. **(B)**Mitochondrial ROS of WT, CHCHD2 knockdown, CHCHD10 knockdown, CHCHD2 and CHCHD10 double knockdown Hela cells. MitoSox was used to determine mitochondrial membrane potential. Error bars are presented as mean ± SD by a one-way ANOVA with Dunnett’s multiple comparisons test (n = 4 independent experiments).  N.S., not significant. **(C)**ATP production of WT, CHCHD2 knockdown, CHCHD10 knockdown, CHCHD2 and CHCHD10 double knockdown Hela cells. Hela cells were cultured in DMEM with 10% FBS and 4.5g/L galactose. (Error bars are presented as mean ± SD by a two-way ANOVA with Tukey’s multiple comparisons test (n = 5 independent experiments), *, p<0.05, **, p<0.01, N.S. not signifcant. **(D)** Mitochondrial membrane potential of WT, CHCHD2 knockdown, CHCHD10 knockdown, CHCHD2 and CHCHD10 double knockdown Hela cells. TMRM was used to determine mitochondrial membrane potential (mean ± SD of n = 4 independent biological samples; one-way ANOVA with Dunnett’s multiple comparisons test. N.S., not significant).

**Supplementary Figure 3**

Lysates of CHCHD2-KO Hela cells expressing exogenous CHCHD2, its mutation T61I or R145Q were immunoprecipitated with anti-CHCHD2, and the protein samples were subjected to immunoblot usig the indicated antibodies.

**Video S1**. Time-Lapse Video of Mitochondrial Fusion and Fission in HeLa Cells, Related to Figure 1 Mitochondria in control HeLa cells expressing mito-DsRed and mito-PA-GFP were photoactivated and tracked by time-lapse confocal imaging for 20 min.

**Video S2**. Time-Lapse Video of Mitochondrial Fusion and Fission in HeLa Cells, Related to Figure 1 Mitochondria in CHCHD2 knockdown HeLa cells expressing mito-DsRed and mito-PA-GFP were photoactivated and tracked by time-lapse confocal imaging for 20 min.

**Video S3**. Time-Lapse Video of Mitochondrial Fusion and Fission in HeLa Cells, Related to Figure 1 Mitochondria in CHCHD10 knockdown HeLa cells expressing mito-DsRed and mito-PA-GFP were photoactivated and tracked by time-lapse confocal imaging for 20 min.

**Video S4**. Time-Lapse Video of Mitochondrial Fusion and Fission in HeLa Cells, Related to Figure 1 Mitochondria in CHCHD2 and CHCHD10 double knockdown HeLa cells expressing mito-DsRed and mito-PA-GFP were photoactivated and tracked by time-lapse confocal imaging for 20 min.
