# Supplementary figures and images for: CHCHD2 and CHCHD10 regulate mitochondrial dynamics and integrated stress response
Source: Cell Death Dis. 2022 Feb 16;13(2):156. doi: 10.1038/s41419-022-04602-5 (PMC8850591; doi:10.1038/s41419-022-04602-5)

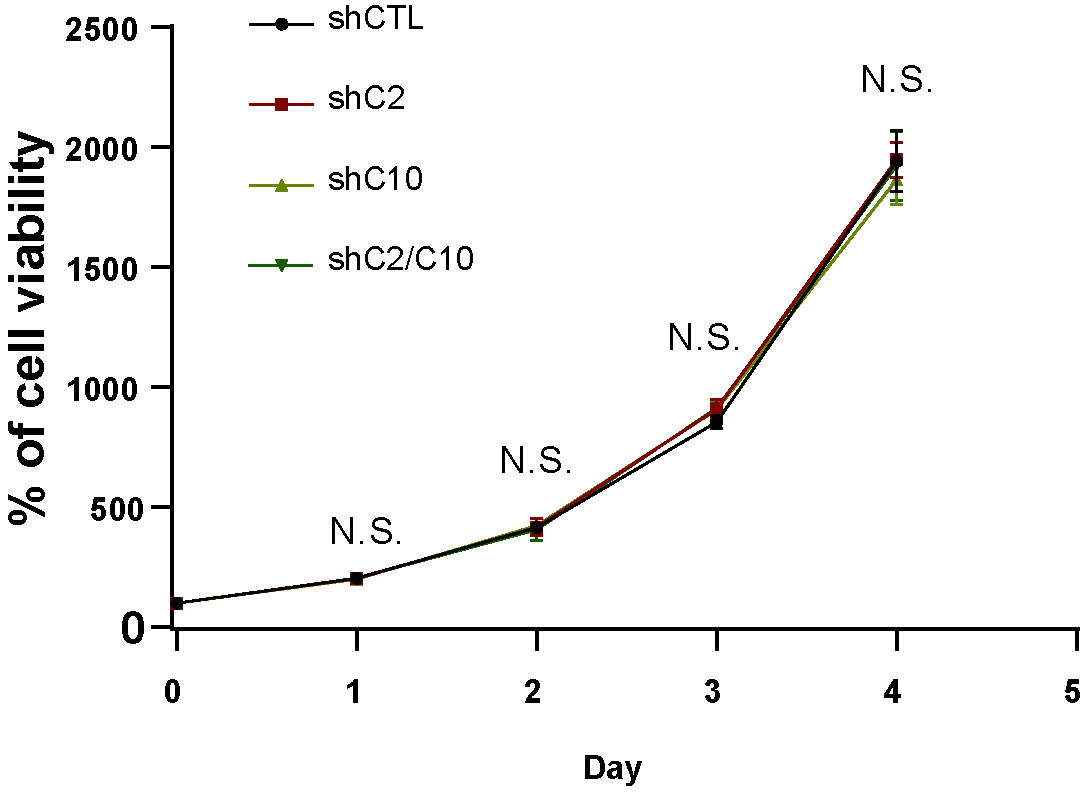

Supplement: Supplementary file 7 — Supplementary Figure 1 [file 41419_2022_4602_MOESM7_ESM.tif]

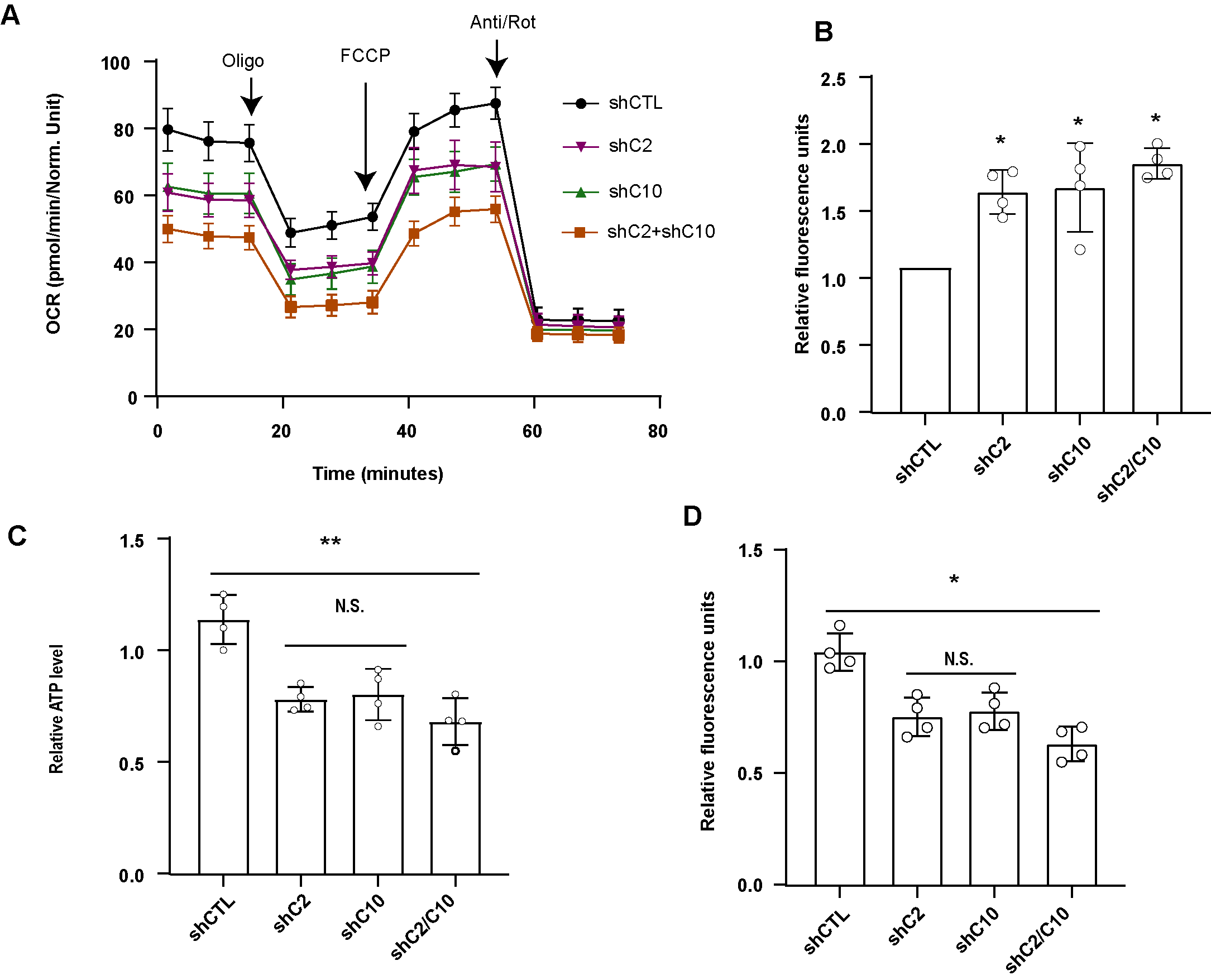

Supplement: Supplementary file 8 — Supplementary Figure 2 [file 41419_2022_4602_MOESM8_ESM.tif]

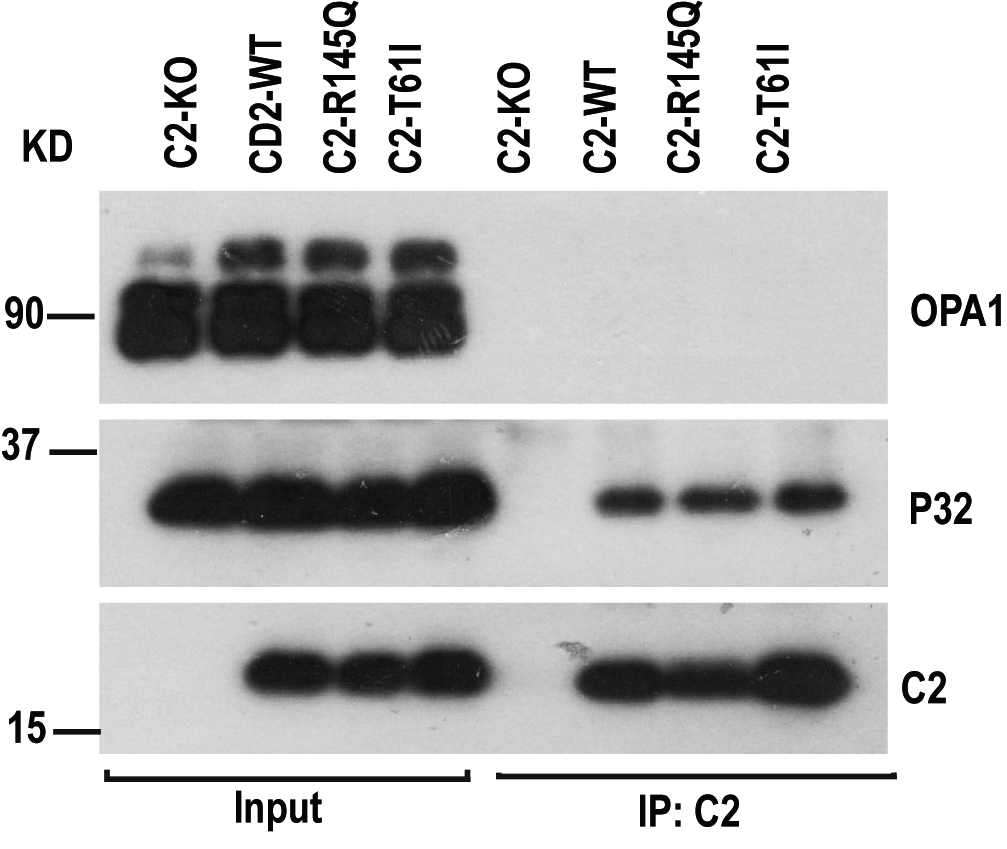

Supplement: Supplementary file 9 — Supplementary Figure 3 [file 41419_2022_4602_MOESM9_ESM.tif]
